# Supplementary material for: Functional comparison of MERS-coronavirus lineages reveals increased replicative fitness of the recombinant lineage 5
Source: Nat Commun. 2021 Sep 7;12:5324. doi: 10.1038/s41467-021-25519-1 (PMC8423819; doi:10.1038/s41467-021-25519-1)
Supplement: Supplementary file 1 — Supplementary information [file 41467_2021_25519_MOESM1_ESM.pdf]

Supplementary information to manuscript “Functional comparison of MERS-coronavirus lineages reveals increased replicative fitness of the recombinant lineage 5”, Schroeder et al., 2021

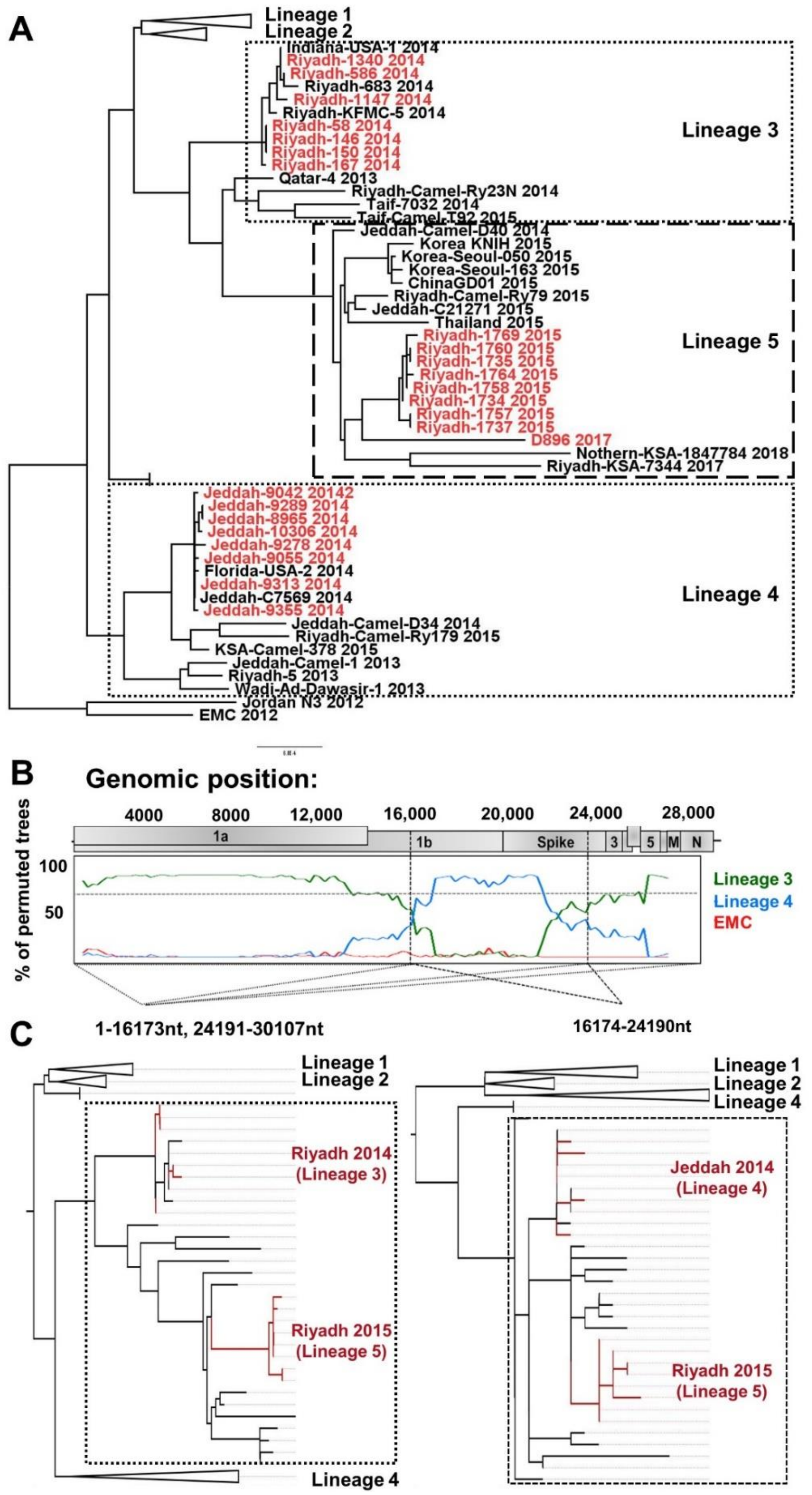

Supplementary Figure 1. MERS-CoV isolates used for this study cluster with three distinct phylogenetic lineages. A) IQtree phylogenetic tree including reference sequences

and coding-complete genomes of virus isolates under study (red). Phylogenetic lineages 1 and 2 are collapsed only to focus the figure. B) Bootscanning recombination analysis based on the coding-complete genome alignment. Riyadh-1764 (lineage 5) was set as query and compared to Riyadh-146 (lineage 3), Jeddah-10306 (lineage 4) and EMC/2012 (outlier) sequences (GenBank accession number JX869059). C) Bayesian phylogenetic tree based on alignments comprising concatenated 5´-proximal and 3´-distal sequences as indicated in the figure (left), as well as the central fragment situated between recombination breakpoints (right). Posterior support values are only shown if below 0.85. Viruses isolated in the present study are highlighted in red.

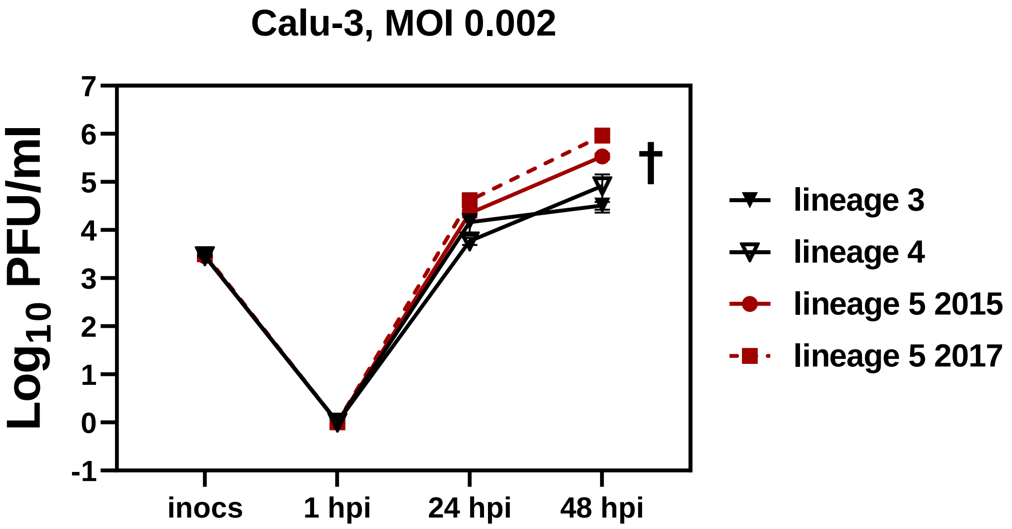

Supplementary Figure 2: Enhanced replication of lineage 5 virus isolates from 2017 on Calu-3 cells. Cells were infected at MOI = 0.002 and virus progeny in supernatant quantified by plaque assay in cell culture supernatants. Infections were performed in duplicates and the experiment was performed twice. Shown are the mean PFU/ml of both experiments.

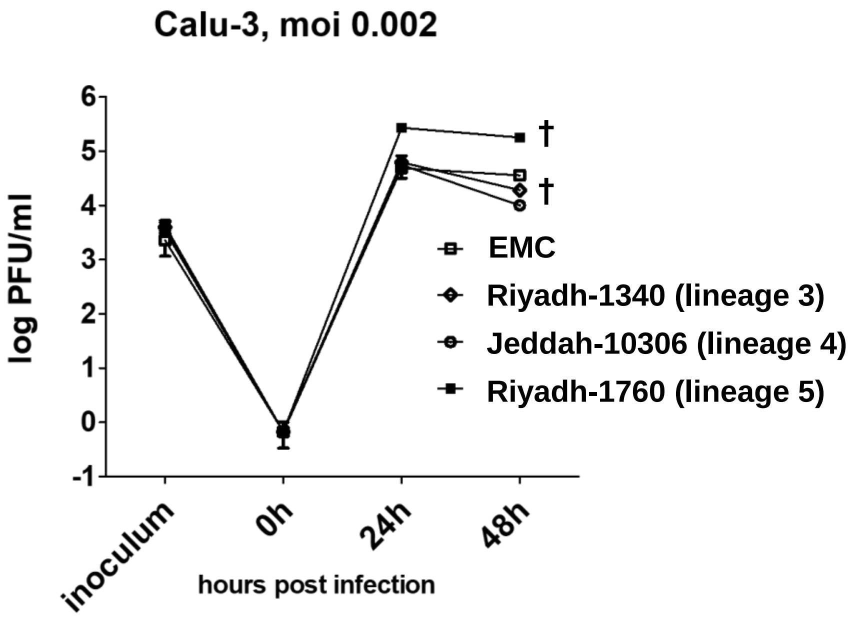

Supplementary Figure 3. 48 hour time course of MERS-CoV multicycle infection (MOI = 0.002) on Calu-3 cells infected with the indicated MERS-CoV isolates. Shown are the PFU/ml of a representative experiment performed with triplicate infections, with error bars indicating SD and the middle line representing the median. The † symbol indicates the complete CPE observed at 48 hpi.

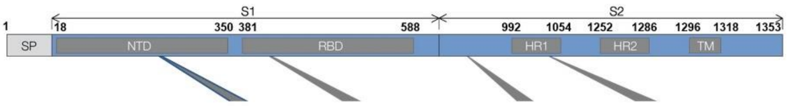

Phylogenetic Lineage	Virus isolate	228	411	833	1020
	EMC	F	L	Q	Q
Lineage 3	Riyadh-58 2014	·	F	·	R
Lineage 3	Riyadh-146 2014	·	F	·	R
Lineage 3	Riyadh-150 2014	·	F	·	R
Lineage 3	Riyadh-167 2014	·	F	·	R
Lineage 3	Riyadh-586 2014	·	F	·	R
Lineage 3	Riyadh-1147 2014	·	F	·	R
Lineage 3	Riyadh-1340 2014	·	F	·	R
Lineage 5	Riyadh-1734 2015	·	·	·	R
Lineage 5	Riyadh-1735 2015	·	·	·	R
Lineage 5	Riyadh-1737 2015	·	·	·	R
Lineage 5	Riyadh-1757 2015	·	·	·	R
Lineage 5	Riyadh-1758 2015	·	·	·	R
Lineage 5	Riyadh-1760 2015	·	·	·	R
Lineage 5	Riyadh-1764 2015	·	·	·	R
Lineage 5	Riyadh-1769 2015	·	·	·	R
Lineage 5	D896 2017	L	·	·	R
Lineage 4	Jeddah-8965 2014	·	·	R	R
Lineage 4	Jeddah-9042 2014	·	·	R	R
Lineage 4	Jeddah-9055 2014	·	·	R	R
Lineage 4	Jeddah-9278 2014	·	·	R	R
Lineage 4	Jeddah-9289 2014	·	·	R	R
Lineage 4	Jeddah-9313 2014	·	·	R	R
Lineage 4	Jeddah-9355 2014	·	·	R	R
Lineage 4	Jeddah-10306 2014	·	·	R	R

Supplementary Figure 4. Amino acid polymorphisms in the Spike protein of the MERS-CoV isolates used in this study. SP = signal peptide, NTD = N-terminal domain, RBD = receptor-binding domain, HR = heptad repeat, TM = transmembrane domain.

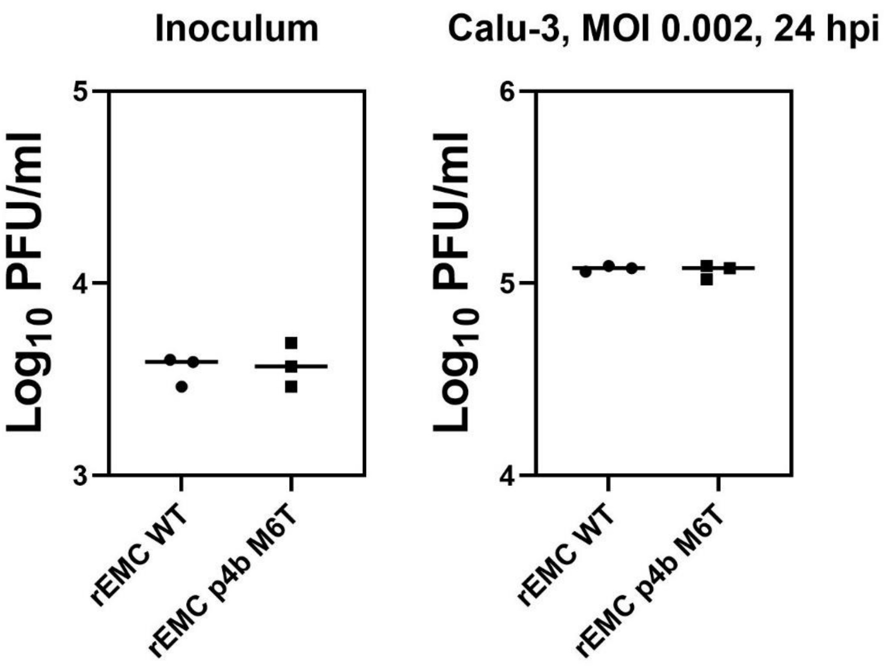

Supplementary Figure 5. The M6T mutation in p4b does not correlate with increased virus replication. Calu-3 cells were infected with rEMC WT and rEMC p4b M6T at an MOI = 0.002 and virus progeny was quantified by plaque titration at 24 hpi. Infections were performed in triplicates and the experiment was performed three times. Shown are the means of each experiment, with bars indicating the mean of all three experiments.

Supplementary Figure 6. Gating strategy used for DPP4 binding assay (Figure 4). Shown here for MERS-CoV wild-type (wt) Spike and control cells using the 1:50 dilution of soluble DPP4-Fc. The same gating strategy was used for all MERS-CoV Spike constructs and for all soluble DPP4-Fc concentrations.

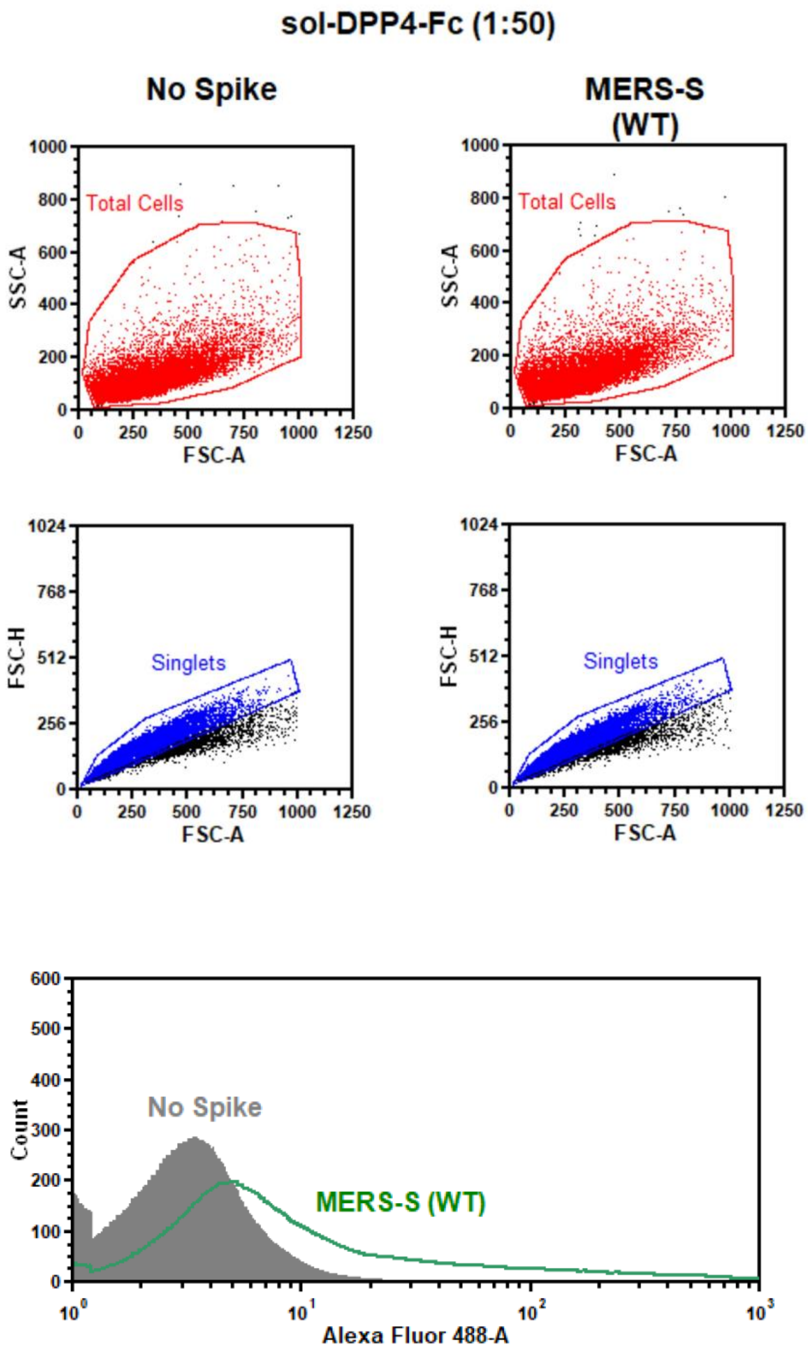

Supplementary Table 1: Summary of MERS-CoV isolates that were used in each experiment and their respective phylogenetic lineage.

Figure	MERS-CoV isolate	lineage
**2A**	Riyadh-146 2014	3
	Riyadh-1147 2014	3
	Jeddah-9278 2014	4
	Jeddah-10306 2014	4
	Riyadh-1734 2015	5
	Riyadh-1764 2015	5
**2B**	Riyadh-58 2014	3
	Riyadh-146 2014	3
	Riyadh-150 2014	3
	Riyadh-167 2014	3
	Riyadh-586 2014	3
	Riyadh-1147 2014	3
	Riyadh-1340 2014	3
	Jeddah-8965 2014	4
	Jeddah-9042 2014	4
	Jeddah-9055 2014	4
	Jeddah-9278 2014	4
	Jeddah-9313 2014	4
	Jeddah-9355 2014	4
	Jeddah-9355 2014	4
	Jeddah-10306 2014	4
	Riyadh-1734 2015	5
	Riyadh-1735 2015	5
	Riyadh-1737 2015	5
	Riyadh-1757 2015	5
	Riyadh-1758 2015	5
	Riyadh-1760 2015	5
	Riyadh-1764 2015	5
	Riyadh-1769 2015	5
**2C**	Riyadh-146 2014	3
	Riyadh-1147 2014	3
	Jeddah-9278 2014	4
	Jeddah-10306 2014	4
	Riyadh-1734 2015	5
	Riyadh-1764 2015	5
**2D**	Riyadh-1147 2014	3
	Jeddah-10306 2014	4
	Riyadh-1764 2015	5

Figure	MERS-CoV isolate	Lineage
**3**	Riyadh-1147 2014	3
	Riyadh-1764 2015	5
**4**	VSVpp	3
	VSVpp	4
	VSVpp	5
**5**	Riyadh-1147 2014	3
	Jeddah-10306 2014	4
	Riyadh-1764 2015	5
**6**	Riyadh-1147 2014	3
	Jeddah-10306 2014	4
	Riyadh-1734 2015	5
**7**	Riyadh-146 2014	3
	Riyadh-1147 2014	3
	Jeddah-9289 2014	4
	Jeddah-10306 2014	4
	Riyadh-1734 2015	5
	Riyadh-1737 2015	5
**8**	Riyadh-146 2014	3
	Riyadh-167 2014	3
	Jeddah-9278 2014	4
	Jeddah-9289 2014	4
	Riyadh-1734 2015	5
	Riyadh-1737 2015	5
**9**	Riyadh-1147 2014	3
	Riyadh-146 2014	3
	Jeddah-8965 2014	4
	Jeddah-9278 2014	4
	Riyadh-1764 2015	5
	Riyadh-1734 2015	5
**10A and C**	Riyadh-1147 2014	3
	Riyadh-146 2014	3
	Jeddah-10306 2014	4
	Jeddah-9278 2014	4
	Riyadh-1764 2015	5
	Riyadh-1734 2015	5
**10B,D and E**	Riyadh-1147 2014	3
	Jeddah-10306 2014	4
	Riyadh-1764 2015	5

Supplementary Table 2. Plaque reduction neutralization assays (PRNT50 dilutions) with five sera for four MERS-CoV isolates. KSA = Kingdom of Saudi Arabia

**Reference serum designation**	**Virus strain***			
	**EMC/2012**	**Riyadh-1147 (lineage 3)**	**Jeddah-9313 (lineage 4)**	**Riyadh-1732 (lineage 5)**
**Munich-1 (MERS patient, Germany 2014) (1)**	1:1280	1:1280	1:1280	1:1280
**SA278 (MERS patient, KSA 2014) (2)**	1:2560	1:2560	1:2560	1:2560
**Dubai-S1 (dromedary camel, UAE 2014) (3)**	1:10240	1:10240	1:10240	1:10240
**Kenia-ILRI (dromedary camel, Kenya 2017) (4)**	1:5120	1:5120	1:5120	1:5120
**Pakistan-493 (dromedary camel, Pakistan 2015) (5)**	> 1:10240	> 1:10240	> 1:10240	> 1:10240

*indicated viruses were neutralized with two human sera and three dromedary camel sera as indicated. The data indicate serum dilutions at which 50% of plaque forming units are neutralized as compared to control.

Supplementary Table 3: Amino acid divergence of MERS-CoV lineages used in the study. The table summarizes non-silent/coding mutations in each viral protein of each respective phylogenetic lineage. Amino acid exchanges found exclusively in all 2015 and the 2017 lineage 5 isolate are highlighted in bold. PLPro domain in nsp3 is highlighted in italics. The M6T mutation that we studied using our reverse genetics system is underlined. aa = amino acid; nsp = non-structural protein; p = protein; M = membrane protein; N = nucleocapsid protein. An overview of amino acid divergence in the spike protein is provided separately in Supplementary Figure 4.

Protein	aa position	EMC	Lineage 3	Lineage 4	Lineage 5 2015	Lineage 5 2017
nsp1	8	T	I	T	I	I
**nsp1**	**158**	**F**	**F**	**F**	**V**	**V**
nsp2	26	K	E	K	K	K
nsp2	196	A	V	A	A	A
**nsp2**	**326**	**L**	**L**	**L**	**I**	**I**
nsp3	34	T	T	T	T	I
nsp3	147	T	V	I	V	V
**nsp3**	**187**	**A**	**A**	**A**	**V**	**V**
**nsp3**	**192**	**A**	**A**	**A**	**V**	**V**
nsp3	219	Q	Q	Q	R	Q
nsp3	257	P	L	P	P	P
nsp3	349	V	V	V	V	I
**nsp3**	**383**	**A**	**A**	**A**	**T**	**T**
nsp3	768	H	H	H	H	Y
nsp3	*847*	*R*	*C*	*R*	*R*	*R*
nsp3	*941*	*P*	*P*	*S*	*P*	*P*
nsp3	*963*	*E*	*E*	*E*	*E*	*D*
**nsp3**	**982**	**E**	**E**	**E**	**A**	**A**
nsp3	1111	E	A	E	A	A
**nsp3**	**1150**	**A**	**A**	**A**	**V**	**V**
**nsp3**	**1266**	**M**	**M**	**M**	**I**	**I**
nsp3	1362	K	K	K	E	K
**nsp3**	**1573**	**T**	**T**	**T**	**I**	**I**
**nsp4**	**7**	**A**	**A**	**A**	**V**	**V**
nsp4	299	A	A	A	A	V
nsp6	232	L	L	F	L	L
nsp8	19	Q	Q	Q	Q	R
**nsp10**	**100**	**A**	**A**	**A**	**V**	**V**
nsp13	364	S	N	S	S	S
**nsp14**	**49**	**V**	**V**	**V**	**I**	**I**
nsp14	122	V	V	V	V	F
nsp14	460	I	I	I	V	I
nsp14	473	A	T	A	A	A
**nsp15**	**148**	**M**	**M**	**M**	**I**	**I**
**p3**	**17**	**L**	**L**	**L**	**F**	**F**

p3	85	G	G	G	L	G
**p4b**	**6**	**M**	**M**	**M**	**T**	**T**
p4b	47	F	F	L	L	L
p4b	85	F	F	L	L	Y
**M**	**123**	**F**	**F**	**F**	**I**	**I**
M	144	T	P	P	P	T
N	11	S	S	S	S	F
N	126	D	D	H	D	D
N	144	S	L	S	S	S
N	283	L	L	L	F	L

Supplementary Table 4: GenBank accession numbers and hyperlinks of MERS-CoV isolates used in this study

Name	Collection date	Phylogenetic clade and lineage	GenBank accession number	hyperlink
EMC-2012	2012	A	JX869059	https://www.ncbi.nlm.nih.gov/nuccore/JX869059
Riyadh_58_2014	2014	B, lineage 3	MN481964	https://www.ncbi.nlm.nih.gov/nuccore/MN481964
Riyadh_150_2014	2014	B, lineage 3	MN481965	https://www.ncbi.nlm.nih.gov/nuccore/MN481965
Riyadh_146_2014	2014	B, lineage 3	MN481966	https://www.ncbi.nlm.nih.gov/nuccore/MN481966
Riyadh_1734_2015	2015	B, lineage 5	MN481979	https://www.ncbi.nlm.nih.gov/nuccore/MN481979
Riyadh_1147_2014	2014	B, lineage 3	MN481967	https://www.ncbi.nlm.nih.gov/nuccore/MN481967
Riyadh_586_2014	2014	B, lineage 3	MN481968	https://www.ncbi.nlm.nih.gov/nuccore/MN481968
Riyadh_1735_2015	2015	B, lineage 5	MN481980	https://www.ncbi.nlm.nih.gov/nuccore/MN481980
Riyadh_1737_2015	2015	B, lineage 5	MN481981	https://www.ncbi.nlm.nih.gov/nuccore/MN481981
Riyadh_1340_2014	2014	B, lineage 3	MN481969	https://www.ncbi.nlm.nih.gov/nuccore/MN481969

Riyadh_1760_2015	2015	B, lineage 5	MN481982	https://www.ncbi.nlm.nih.gov/nuccore/MN481982
Riyadh_1758_2015	2015	B, lineage 5	MN481983	https://www.ncbi.nlm.nih.gov/nuccore/MN481983
Riyadh_1757_2015	2015	B, lineage 5	MN481984	https://www.ncbi.nlm.nih.gov/nuccore/MN481984
Riyadh_1769_2015	2015	B, lineage 5	MN481985	https://www.ncbi.nlm.nih.gov/nuccore/MN481985
Jeddah_9042_2014	2014	B, lineage 4	MN481970	https://www.ncbi.nlm.nih.gov/nuccore/MN481970
Jeddah_9055_2014	2014	B, lineage 4	MN481971	https://www.ncbi.nlm.nih.gov/nuccore/MN481971
Jeddah_9278_2014	2014	B, lineage 4	MN481972	https://www.ncbi.nlm.nih.gov/nuccore/MN481972
Jeddah_8965_2014	2014	B, lineage 4	MN481973	https://www.ncbi.nlm.nih.gov/nuccore/MN481973
Jeddah_9313_2014	2014	B, lineage 4	MN481974	https://www.ncbi.nlm.nih.gov/nuccore/MN481974
Riyadh_1764_2015	2014	B, lineage 5	MN481986	https://www.ncbi.nlm.nih.gov/nuccore/MN481986
Jeddah_9289_2014	2014	B, lineage 4	MN481975	https://www.ncbi.nlm.nih.gov/nuccore/MN481975
Riyadh_167_2014	2014	B, lineage 3	MN481976	https://www.ncbi.nlm.nih.gov/nuccore/MN481976

Jeddah_9355_2014	2014	B, lineage 4	MN481977	https://www.ncbi.nlm.nih.gov/nuccore/MN481977
Jeddah_10306_2014	2014	B, lineage 4	MN481978	https://www.ncbi.nlm.nih.gov/nuccore/MN481978

Supplementary Table 5: Oligonucleotides used in this study.

Primer name:	Sequence (5´-3´):
S:Q1020R F	TAATGAAGCTTTTCGGAAGGTTCAGGATGC
S:Q1020R F	GTTGTAGTGAAGCCTGTTTGCATAGCTCCC
S:L411 F	CCAATTGCAATTATAATTTTACCAAATTGCTTTCACTTTTTTCTGT
S:L411 R	TAAAAACCAAACGCTTGAAATTATAAACCTGAGGAGGT
S:Q833R F	TTCCAAAATAAACCGGGCTCTCCATGGTGC
S:Q833R R	CAAAACTGGCCATACTCGCGCAGTAATTGC
Amplicon 1 F	TACCTGGTTGAGAGGCTCAT
Amplicon 1 R	CTTAAGCAGATTCTGGGCATATT
Amplicon 2 F	TGAGTGTGGAAGTTGTGGTAAT
Amplicon 2 R	ACCTTTGAGAAGCTGGCGTATT
Amplicon 3 F	TCGAGCCGCATAAGGTTCAT
Amplicon 3 R	GCTGAGCTGCGTCCTGTTT
TBP F	CTGCGGTAATCATGAGGATAAG
TBP prb	TGTGCACAGGAGCCAAGAGTGAAG
TBP R	TTCTTGCTGCCAGTCTGGAC
IFNB1 F	AGGATTCTGGATTACCTGAAGG
IFNB1 prb	TCCACTCTGACTATGGTCCAGGCA
IFNB1 R	GGCTAGGAGATCTTCAGTTTCG
IFNL1 F	CTCTGTCACCTTCAACCTCTTC
IFNL1 prb	CACGCGAGACCTGAATTGTGTTGC
IFNL1 R	ATCTCAGGTTGCATGACTGG
CCL5 F	TGCCCACATCAAGGAGTATTTC
CCL5 prb	TCACCCGAAAGAACCGCCAAGT
CCL5 R	CCATCCTAGCTCATCTCCAAAG
MX1 F	TTCAGCACCTGATGGCCTATC
MX1 prb	CAGGAGGCCAGCAAGCGCCATC
MX1 R	TGG ATG ATC AAA GGG ATG TGG
TNFa F	TGGCCCAGGCAGTCAGA
TNFa prb	CATCTTCTCGAACCCCGAGTGACAAGC
TNFa R	TGTAGCCCATGTTGTAGCAAACC
sgmRNA N F (6)	CTCGTTCTCTTGCAGAACTTTG
sgmRNA N prb (6)	CACGAGCTGCACCAAATAACACTGTCTC
sgmRNA N R (6)	GTAAGAGGGACTTTCCCGTGTTG
MERS ORF1a F (7)	CCACTACTCCCATTTCGTCAG
MERS ORF1a prb (7)	CAGTATGTGTAGTGCGCATATAAGCA
MERS ORF1a R (7)	TTGCAAATTGGCTTGCCCCCACT

References

1. Drosten C, Seilmaier M, Corman VM, Hartmann W, Scheible G, Sack S, et al. Clinical features and virological analysis of a case of Middle East respiratory syndrome coronavirus infection. The Lancet Infectious Diseases. 2013;13(9):745-51.
2. Muth D, Corman VM, Meyer B, Assiri A, Al-Masri M, Farah M, et al. Infectious Middle East Respiratory Syndrome Coronavirus Excretion and Serotype Variability Based on Live Virus Isolates from Patients in Saudi Arabia. J Clin Microbiol. 2015;53(9):2951-5.
3. Meyer B, Müller MA, Corman VM, Reusken CB, Ritz D, Godeke GJ, et al. Antibodies against MERS coronavirus in dromedary camels, United Arab Emirates, 2003 and 2013. Emerg Infect Dis. 2014;20(4):552-9.
4. Corman VM, Jores J, Meyer B, Younan M, Liljander A, Said MY, et al. Antibodies against MERS coronavirus in dromedary camels, Kenya, 1992-2013. Emerg Infect Dis. 2014;20(8):1319-22.
5. Saqib M, Sieberg A, Hussain MH, Mansoor MK, Zohaib A, Lattwein E, et al. Serologic Evidence for MERS-CoV Infection in Dromedary Camels, Punjab, Pakistan, 2012-2015. Emerg Infect Dis. 2017;23(3):550-1.
6. Zhou J, Li C, Zhao G, Chu H, Wang D, Yan HH-N, et al. Human intestinal tract serves as an alternative infection route for Middle East respiratory syndrome coronavirus. Science Advances. 2017;3(11):eaao4966.
7. Corman VM, Eckerle I, Bleicker T, Zaki A, Landt O, Eschbach-Bludau M, et al. Detection of a novel human coronavirus by real-time reverse-transcription polymerase chain reaction. Euro Surveill. 2012;17(39).
